# Long-acting family planning uptake and associated factors among women in the reproductive age group in East Africa: multilevel analysis

**DOI:** 10.3389/fgwh.2025.1444784

**Published:** 2025-02-03

**Authors:** Ermias Bekele Enyew, Abiyu Abadi Tareke, Habtamu Setegn Ngusie, Mulugeta Desalegn Kasaye, Shimels Derso Kebede, Mahider Shimelis Feyisa

**Affiliations:** ^1^ Department of Health Informatics, College of Medicine and Health Sciences, Wollo University, Dessie, Ethiopia; ^2^ Amref Health Africa in Ethiopia, West Gondar Zonal Health Department, Gondar, Ethiopia; ^3^ Department of Health Informatics, School of Public Health, College of Medicine and Health Sciences, Woldia University, Woldia, Ethiopia; ^4^ Department of Medical Laboratory, College of Health Science, Debre Tabor University, Debre Tabor, Ethiopia

**Keywords:** prevalence, long-acting contraceptive method, multilevel analysis, east Africa, DHS

## Abstract

**Introduction:**

The campaign to encourage sexually active women to utilize family planning is one of the primary initiatives being undertaken globally to reduce unintended pregnancies and fertility rates. Evidence suggests that family planning measures can lower this maternal mortality ratio by nearly 25%. According to our literature search, there is no known study that has reported on the study area to assess utilization and factors associated with the use of long-acting contraceptive methods (LACMs) among women of reproductive age. Therefore, this study aimed to assess long-acting contraceptive method uptake and its associated factors among women of reproductive age in East Africa.

**Methods:**

A weighted total of 50,525 women of reproductive age were included in this study. A community-based cross-sectional study was conducted on the most recent Demographic and Health Surveys in 12 East African countries. The pooled prevalence of long-acting contraceptive uptake with a 95% confidence interval (CI) was reported and presented in a forest plot for East African countries using STATA version 14.1. Intraclass correlation coefficient, likelihood ratio (LR) test, median odds ratio, and deviance (−2 log-likelihood) values were used for model comparison and fitness. Adjusted odds ratios (AOR) with a 95% CI and *p*-value ≤0.05 in the multilevel logistic model were used to declare significant factors associated with long-acting contraceptive uptake.

**Results:**

The overall prevalence of long-acting contraceptive methods in East African countries was 19.41% (95% CI 19.07%–19.76%). In the multilevel logistic regression analysis, women in the age group of 35–49 years (AOR 1.09, 95% CI 1.06–1.17), women who were married (AOR 1.31, 95% CI 1.10–1.56), and women who were exposed to media (AOR 1.06, 95% CI 1.00–1.13) were significantly associated with LACM uptake. Moreover, living in urban areas (AOR 1.23, 95% CI 1.14–1.32) and living in the highest household wealth index (AOR 1.09, 95% CI 1.01–1.17) were also significantly associated with long-acting contraceptive uptake.

**Conclusion:**

The overall utilization of acting contraceptive methods was low. Therefore, future interventions should be planned to target women in younger age groups, with lower socioeconomic backgrounds, and those living in rural areas to improve LACM uptake.

## Introduction

Family planning (FP) is an essential aspect of public health initiatives in low-income countries as well as global development support programs (1). Women with high-fertility have faced enormous difficulties in meeting their unmet contraceptive needs, which could be due to a lack of understanding about how to protect their health, a lack of service, or a lack of decision-making power in post-pondering childbearing (2). The campaign to encourage sexually active women to utilize family planning is one of the primary initiatives being undertaken globally to reduce unintended pregnancies and lower fertility rates (3, 4). Complications throughout pregnancy and childbirth account for 99% of maternal deaths worldwide, making them the top cause of mortality in low- and middle-income countries. Evidence suggests that family planning measures can lower this maternal mortality ratio by nearly 25% (5).

Globally, in 2019, 45.2% of contraceptive users used long-acting or permanent methods, 46.1% used short-acting methods, and 45.2% used traditional methods (6). In sub-Saharan Africa, the average prevalence is approximately 5%, influenced by cultural beliefs and limited access to healthcare (7, 8). High fertility rates are a feature of the East African region; nations such as Uganda record total fertility rates of 5.4 children per woman, which is higher than the target fertility rate of 4.3 (9). For instance, approximately 5% of women in Uganda (10) and 6% of women in Tanzania use long-acting contraceptive methods (LACMs) (11). In addition, government activities have increased uptake in Ethiopia to approximately 13%, while successful public health campaigns have led to Rwanda having the highest prevalence in the region at nearly 27% (12).

Numerous studies indicate that sociodemographic characteristics, including women's age, marital status, education, domicile, and religion, all influence the adoption of long-active family planning (LAFP) in different contexts (13, 14). Reproductive health characteristics, such as desire for FP, parity, and fertility goal, have also been found in several studies to be associated with LAFP use in general (15, 16). In other studies, reproductive health factors associated with long-acting family planning use included parity, desired family size, women who gave birth prematurely, a previous history of abortion, women who had ever experienced an unwanted pregnancy, and women who had visited a clinic in the previous year for FP services (17–19).

Improving family planning in the area requires addressing the obstacles to the uptake of LACMs. For this reason, numerous scholars have pointed out that promoting voluntary access to a wide variety of contraceptive methods for women is a crucial part of countries’ strategies to advance social and economic development (20, 21). The Sustainable Development Goal (SDG) plans to ensure universal access to sexual and reproductive healthcare services, including for FP, information and education, and the integration of reproductive health into national strategies and program specifically universal access to FP services to ensure healthy lives and wellbeing (22).

Increasing the use of LACMs will improve the general sexual and reproductive health of women in the reproductive age group by lowering the high rates of induced abortion and unwanted pregnancy (23). The proportion of long-acting contraceptive method users is extremely low in high-fertility countries, even though family planning methods can improve the health of mothers and children in countries with high birth rates and nations with limited resources (24). According to our literature search, there is no known study that has reported on the study area to assess utilization and factors associated with the use of long-acting contraceptive method among women of reproductive age. Therefore, this study aimed to assess the uptake of long-acting contraceptives method uptake and its associated factors among women of reproductive age in East Africa. The finding of the study is an input for policymakers and healthcare providers, contributing to global reproductive health initiatives aimed at improving maternal health and achieving sustainable development goals.

## Methods and materials

### Data source, sampling procedures, and study population

The study was conducted based on the most recent Demographic and Health Surveys (DHS) in 12 East African countries (Burundi, Ethiopia, Comoros, Uganda, Rwanda, Tanzania, Mozambique, Madagascar, Zimbabwe, Kenya, Zambia, and Malawi) conducted between 2008 and 2018. These datasets were combined to determine the pooled prevalence and factors associated with long-acting contraceptive method among women of reproductive age in East Africa. The data were downloaded from the https://dhsprogram.com/data/available-datasets.cfm. The DHS used two stages of stratified sampling technique to select the study participants. The key demographic and health indicators were collected in each DHS (25). A weighted total of 50,525 women in the reproductive age group were included in this study, with a complete answer to all factors of interest. Each country's survey report has further details about the data collection technique.

### Variables of the study

The outcome variable for this study was contraceptive use. For this analysis, contraceptive use was grouped into two categories: using long-acting and permanent contraceptive methods [intrauterine devices (IUDs), female sterilization, and implants], coded as “1”; and using other methods (short-acting and traditional), coded as “0.” The independent variables considered for this study were from two sources: individual-level characteristics and community-level characteristics. These variables were chosen based on the review of different literature about factors affecting long-acting contraceptive method uptake. Individual-level factors included maternal age, maternal educational status, paternal education status, maternal occupation, parity, marital status, fertility preference, pregnancy termination, health facility visit, wealth index, and media exposure. Community-level factors included community women's education (low and high levels of maternal literacy), community poverty status (low and high levels of poverty), community media exposure (low and high levels of media exposure), residence (urban, rural), distance from the health facility (long and short distance), and countries.

### Operational definition

#### Community-level poverty

The proportion of women who were from households belonging to the categories of poorest and poorer wealth index. Those who fell in the median value and above were categorized under the high poverty level and those who fell below the median value of the variables were categorized under the low poverty level.

#### Community-level literacy

The proportion of mothers who completed primary school and above were categorized as literate, while mothers who did not complete primary school were categorized as illiterate.

#### Community-level media exposure

The proportion of women in the cluster who had at least some exposure to television, radio, or newspapers was categorized as media exposure, while mothers who did not have at least some exposure to television, radio, or newspapers were categorized as no media exposure.

### Data management and analysis

The statistical software STATA version 14 was used to handle and analyze the data. Before any statistical analysis, the data were weighted to restore the data's representativeness and provide a reliable estimate and standard error. Frequencies and percentages were used to create descriptive statistics. The pooled prevalence of long-acting contraceptive method with a 95% confidence interval (CI) was reported and presented in a forest plot of East African countries.

The DHS data had a hierarchical nature that could violate the independence of observations and the equal variance assumption of the traditional logistic regression model. This implies that there is a need to consider the between-cluster variability by using advanced models. Therefore, a multilevel logistic regression model (both fixed and random effects) was fitted. Since the outcome variable was binary, standard logistic regression and multilevel logistic regression models were fitted.

### Model building

In the multilevel logistic regression model, we fit four models, the null model (model I) without explanatory variables, model II with only individual-level variables, model III with only community-level variables, and model IV with both individual-level and community-level variables. These models were fitted using a STATA command *melogit*. Model comparison and fitness were made based using the intraclass correlation coefficient (ICC), likelihood ratio (LR) test, median odds ratio (MOR), and deviance [−2 log-likelihood (LLR)], Akaki Information Criteria (AIC), and Bayesian Information Criteria (BIC) values since the models were nested. Accordingly, model III (individual + community) were the best-fit model for this study.

### Ethics approval and consent to participate

This study is a secondary data analysis based on DHS data. For this analysis, we registered and requested data for analysis from the DHS online archive. For DHS data, ethical approval has been obtained from the individual national institution's review board and by ICF International Institutional Review Board to download the identified demographic health survey datasets from the DHS Program website (http://www.measuredhs.com). Procedures for DHS public-use datasets certified by the Institutional Review Board do not allow respondents, households, or sample communities to be identifiable in any way.

## Results

### Sociodemographic characteristics of the respondents

A total of 50,525 reproductive age group women were enrolled in this study. Of the women, 51.20% were in the age group of 25–34 years. Regarding maternal educational status, 53.67% had a primary school education and 4.75% had a tertiary level of education. The majority (80.86%) of the women were married and 76.02% had to visit health facilities. In terms of occupation, 68.91% of women were currently employed (Table 1).

**Table 1:** Individual characteristics of reproductive-aged women in East African countries (*n* = 50,525).

Variables	Weighted frequency	Percentage
Age (years)		
15–24	13,798	27.30
25–34	25,876	51.20
35–49	10,869	21.50
Maternal educational status		
No education	8,099	16.03
Primary education	27,120	53.67
Secondary education	12,932	25.58
Tertiary education	2,385	4.75
Husband education status		
No education	6,907	4.06
Primary education	24,497	49.87
Secondary education	13,997	28.49
Tertiary education	3,720	7.57
Maternal working status		
Not working	15,702	31.09
Working	34,810	68.91
Wealth index		
Poor	18,228	36.08
Middle	10,117	20.02
Rich	22,181	43.90
Marital status		
Single	8,554	16.93
Married	40,856	80.86
Widowed/divorced	1,116	2.21
Parity		
1–2 children	19,021	37.65
3–4 children	17,114	33.87
≥5 children	14,391	28.48
Media exposure		
Not exposed	14,042	27.79
Exposed	36,483	72.21
Health facility visit		
No	12,115	23.98
Yes	38,410	76.02
Fertility preference		
Want more	28,014	55.47
Undecided	1,443	2.86
Want no more	21,046	41.67
Pregnancy termination		
No	44,098	87.28
Yes	6,428	12.72

Of the total, 16.02% and 16.73% of the women were from Kenya and Malawi, respectively. The majority 36,888 (73.03%) of the women were rural residents and nearly 63% have distanced from health facility, not big problem (Table 2).

**Table 2:** Community-level characteristics of reproductive-aged women in East African countries (*n* = 50,525).

Variables	Weighted frequency	Percentage
Countries		
Burundi	6,373	12.61
Ethiopia	3,088	6.11
Kenya	8,093	16.02
Comoros	1,104	2.19
Madagascar	3,825	7.57
Malawi	8,111	16.73
Mozambique	1,071	2.12
Rwanda	3,398	6.73
Tanzania	3,146	6.23
Uganda	4,626	9.16
Zambia	3,945	7.81
Zimbabwe	3,740	7.40
Community-level poverty		
Low level	26,314	52.08
High level	24,211	47.92
Community-level women literacy		
Low level	27,165	53.77
High level	23,360	46.23
Community-level media exposure		
Low	24,991	49.46
High	25,534	50.54
Distance from health facility		
Big problem	18,093	37.23
Not big problem	30,503	62.77
Residency		
Urban	13,621	26.97
Rural	36,888	73.03

### Prevalence of long-acting contraceptive uptake among reproductive age group women in East African countries

**Figure 1:**
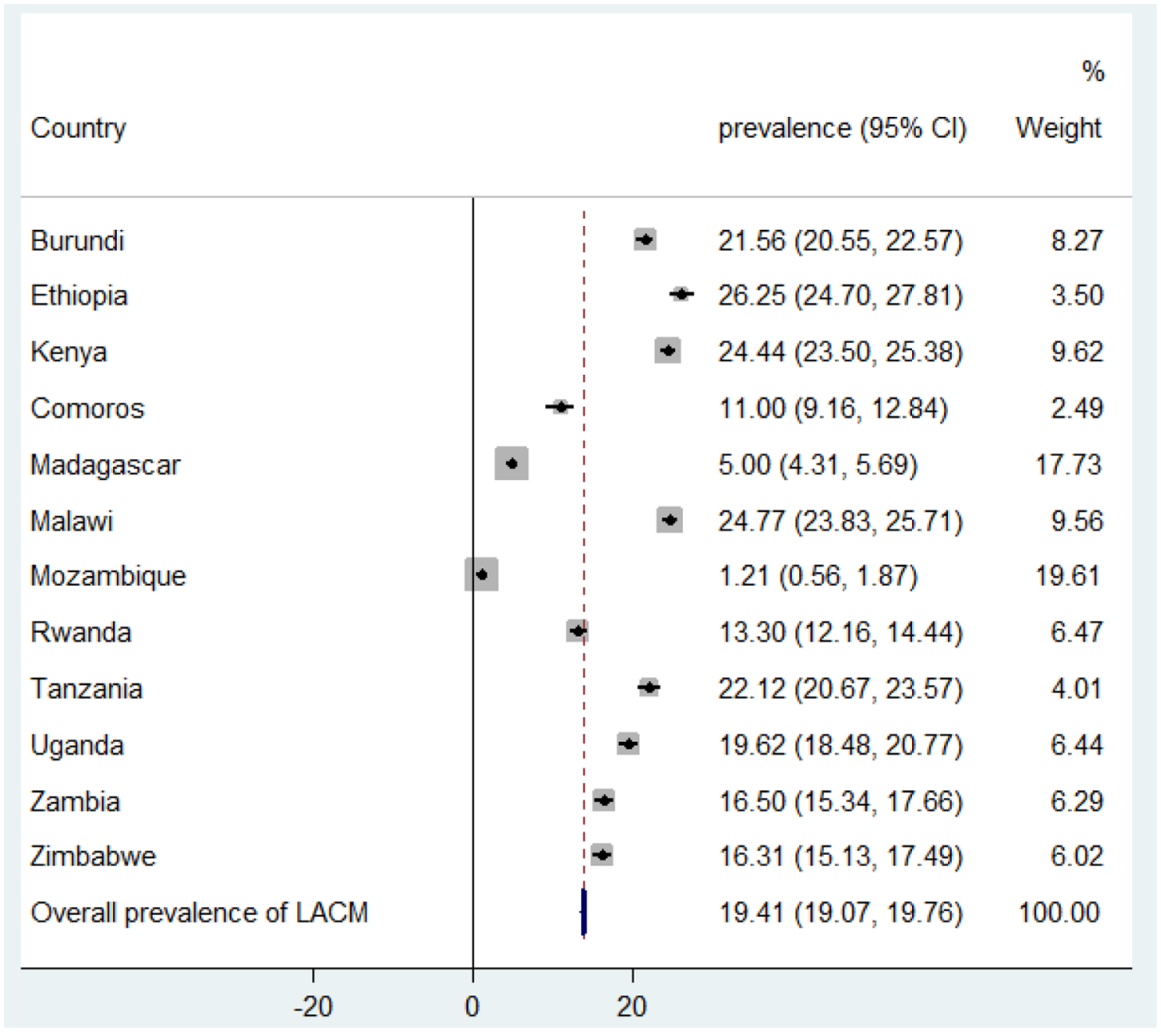
Forest plot of long-acting contraceptive uptake among reproductive-aged women in East Africa.

The pooled prevalence of long-acting contraceptive uptake in East African countries was 19.41% (95% CI 19.07%–19.76%) with a lower proportion of them observed in Mozambique at 1.21% (95% CI 0.56%–1.87%). However, a higher proportion was noticed in Ethiopia (26.25%, 95% CI 24.70%–27.81%) and Malawi (24.77%, 95% CI 23.83%–25.71%) (Figure 1).

### Multilevel logistic regression analysis

#### Factors associated with long-acting contraceptive uptake in east Africa

##### Random effects analysis

In the random effects model, the null model, approximately 19.63% of the total variation in long-acting contraceptive uptake occurred at the community level and is attributable to the community-level factor. The highest (53.75%) proportional change in variance (PCV) in the final model (model IV) implies that both individual and community-level factors explained 53.75% of the variation in long-acting contraceptive methods across the regions (Table 3).

**Table 3:** Model comparison and model fitness for multilevel logistic regression analysis.

Parameters	Null model I	Model II	Model III	Model IV
Random effect				
Community variance	0.80 (0.69–0.92)	0.73 (0.63–0.89)	0.63 (0.53–0.75)	0.37 (0.29–0.58)
ICC%	19.63%	19.73%	11.43%	6.4%
MOR	2.33 (2.20–2.48)	2.25 (2.12–2.45)	2.12 (1.99–2.27)	1.78 (1.66–2.06)
PCV%	1%	8.75%	21.25%	53.75%
Model comparison				
AIC	47,979	47,775	45,661	45,848
BIC	47,996	47,951	45,819	45,799
LLR	−23,987	−23,867	−22,812	−22,706
Deviance	47,974	47,334	45,624	45,412

AIC, Akaike's information criterion; BIC, Bayesian information criterion; LLR, log-likelihood; MOR, median odds ratio; ICC, intraclass correlation coefficient; PCV, proportional change in variance.

##### Fixed effect analysis

As shown in Table 4, the model fitness was checked using deviance and log-likelihood. The model with the highest log-likelihood (−22,706) and the lowest deviance (45,412) (model IV) was the best-fit model; therefore, the fixed effects were interpreted utilizing model IV. In the multivariable mixed-effect binary logistic regression analysis, maternal age, maternal and paternal education status, marital status, maternal occupation status, wealth index, media exposure, fertility preference, place of residence, distance from the health facility, and living countries were significant determinants of long-acting contraceptive method in East African countries.

**Table 4:** Multivariable multilevel logistic regression analysis of both individual and community-level factors associated with long-acting contraceptive methods in East African countries.

Characteristics	Model I	Model II AOR (95% CI)	Model III AOR (95% CI)	Model IV AOR (95% CI)
Maternal age (years)				
15–24		1		1
25–34		0.89 (0.81–0.98)***		0.89 (0.80–0.98)***
35–49		1.13 (1.06–1.21)**		1.09 (1.02–1.17)**
Maternal education				
No education		1		1
Primary		0.91 (0.84–0.98)**		0.97 (0.90–1.06)
Secondary		0.85 (0.78–0.94)**		0.99 (0.89–1.10)
Tertiary		1.36 (1.17–1.97)***		1.48 (1.26–1.74)***
Husband education				
No education		1		1
Primary		0.99 (0.92–1.07)		0.97(0.90–1.06)
Secondary		0.99 (0.90–1.08)		0.99(0.89–1.09)
Tertiary		1.37 (1.20–1.56)***		1.29(1.12–1.48)***
Maternal working				
Not working		1		1
Working		1.05 (1.00–1.11)*		1.09 (1.03–1.15)**
Wealth index				
Poor		1		1
Middle		1.09 (1.02–1.17)*		1.06(0.99–1.14)
Rich		1.22 (1.15–1.30)***		1.09(1.01–1.17)**
Marital status				
Single		1		1
Married		0.99 (0.93–1.106)		0.92(0.85–0.99)*
Widowed/divorced		1.25 (1.06–1.47)**		1.07(0.71–1.48)
Parity				
1–2 children		1		1
3–4 children		1.01 (0.95–1.08)		1.06(0.99–1.32)
≥5 children		0.95 (0.87–1.04)		1.02(0.93–1.12)
Media exposure				
Not exposed		1		1
Exposed		0.96 (0.90–1.02)		1.06(1.00–1.13)*
Health facility visit				
No		1		1
Yes		0.96 (0.91–1.01)		0.86 (0.81–0.91)**
Fertility preference				
Want more		1		1
Undecided		1.11 (0.96–1.28)		1.08 (0.94–1.25)
Want no more		1.10 (1.04–1.17)**		1.08 (1.02–1.15)**
Pregnancy termination				
Yes		1		1
No		1.23 (1.14–1.33)*		1.23 (0.97–1.33)
Community-level factors				
Place of residency				
Rural			1	1
Urban			1.37 (1.28–1.48)**	1.23 (1.14–1.32)***
Perceived distance from health facility				
Big problem			1	1
Not big problem			1.07 (1.02–1.13)**	1.06 (1.00–1.12)*
Community-level poverty				
Low level			1	1
High level			0.96 (0.84–1.08)	0.98 (0.87–1.12)
Community-level literacy				
Low level			1	1
High level			0.99 (0.87–1.13)	0.98 (0.86–1.12)
Community-level media exposure				
Low level			1	1
High level			0.91 (0.80–1.04)	0.90 (0.79–1.03)
Country				
Burundi			1	1
Ethiopia			1.34 (1.19–1.50)**	1.36 (1.20–1.53)***
Kenya			1.05 (0.95–1.17)	1.01 (0.91–1.13)
Comoros			0.45 (0.36–0.55)**	0.44 (0.36–0.55)***
Madagascar			0.17 (0.14–0.22)**	0.16 (0.13–0.20)***
Malawi			1.22 (1.12–1.34)**	1.20 (1.09–1.33)***
Mozambique			0.03 (0.02–0.06)**	0.03 (0.01–0.06)***
Rwanda			0.51 (0.45–0.57)**	0.49 (0.45–0.56)***
Tanzania			0.93 (0.83–1.04)	0.91 (0.81–1.02)
Uganda			0.88 (0.80–0.98)*	0.83 (0.74–0.93)**
Zambia			0.63 (0.56–0.72)**	0.63 (0.56–0.71)***
Zimbabwe			0.63 (0.56–0.71)**	0.59 (0.52–0.67)***

*P < 0.05; **P < 0.01; ***P < 0.001.

After controlling for other individual and community-level factors, women aged 34–49 years were 1.09 times more likely to use LACMs [adjusted odds ratio (AOR) 1.09, 95% CI 1.06–1.17] compared with women aged 15–24 years. Regarding parental education status, mothers with tertiary education were 1.48 times more likely to use LACMs (AOR 1.48, 95% CI 1.26–1.74) and women with higher educated husbands were 1.29 times more likely (AOR 1.29, 95% CI 1.12–1.48) compared to their less educated counterparts. Women who were currently married were 0.92 times (AOR 0.92, 95% CI 0.85–0.99) less likely to use LACMs than single women. The household wealth index was found to be a predictor of LACM use. Women who were living at the highest level of the household wealth index were 1.09 times (AOR 1.09, 95% CI 1.01–1.17) more likely to use LACMs than women who were at the lowest level of the household wealth index.

Regarding the employment status of women, working women were 1.09 times (AOR 1.09, 95% CI 1.03–1.15) more likely to use LACMs than women who were not working. Women who had no desire for more children were 1.08 times (AOR 1.08, 95% CI 1.02–1.15) more likely to use LACMs than women who wanted more children. Women who were exposed to family planning messages on the radio, TV, and in newspapers or magazines in the past 12 months before the survey were 1.06 times (AOR 1.06, 95% CI 1.00–1.13) more likely to use LACMs than their non-exposed counterparts. However, women who visited health facilities were also 0.86 times (AOR 0.86, 95% CI 0.81–0.91) less likely to use LACMs than those who had not visited health facilities.

Regarding community-level factors, women who lived in urban areas were 1.23 times (AOR 1.23, 95% CI 1.14–1.32) more likely to use LACMs than women who lived in rural areas. In addition, women who were close to the health facility were 1.06 times (AOR 1.06, 95% CI 1.00–1.12) more likely to use LACMs than women who lived further away from the health facility. Women who lived in Comoros, Madagascar, Mozambique, Rwanda, Uganda, Zambia, and Zimbabwe were 0.44 times (AOR 0.44, 95% CI 0.36–0.55), 0.16 times (AOR 0.16, 95% CI 0.13–0.20), 0.03 times (AOR 0.03, 95% CI 0.01–0.06), 0.49 times (AOR 0.49, 95% CI 0.45–0.56), 0.83 times (AOR 0.83, 95% CI 0.74–0.93), 0.63 times (AOR 0.63, 95% CI 0.56–0.71), and 0.59 times (AOR 0.59, 95% CI 0.52–0.67) less likely to use LACMs, respectively, than women who lived in Burundi. On the other hand, women who lived in Ethiopia and Malawi were 1.36 times (AOR 1.36, 95% CI 1.20–1.53) and 1.20 times (AOR 1.20, 95% CI 1.09–1.33) more likely to use LACMs, respectively, than women who lived in Burundi (Table 4).

## Discussion

The study aimed to assess the utilization of LACMs among reproductive-aged women in East African countries, drawing on data from the most recent DHS. The WHO emphasizes that LACMs, including implants and IUDs, are some of the best ways to improve reproductive health and avoid unwanted births. Furthermore, integrating family planning services into maternal healthcare systems is necessary to increase access to and acceptance of LACMs, particularly among women of reproductive age (26).

The findings indicate that the prevalence of LACM use stands at approximately 19.41%, which is significantly lower than the targets set by the SDGs aimed at ensuring universal access to sexual and reproductive health services by 2030 (22). This disparity reveals important obstacles to the adoption of LACM, such as partner support, educational attainment, and sociocultural factors. These findings are in line with research published by the WHO, which emphasizes the significance of family planning in enhancing maternal and child health outcomes (26). This finding is consistent with studies carried out in Kenya (20.6%) (14) and Ethiopia (20.4%) (27). This figure is notably higher than the prevalence rates reported in Nepal (4.7%) (15), Indonesia (16.5% (28), and a localized study conducted in Mekele, Ethiopia (12.0%) (29). Conversely, it is lower than the rates observed in Uganda (31.7%) (30) and the United Kingdom (28%) (31). The variations in study populations, periods, and societal influences are some of the causes of these discrepancies in the prevalence of contraception. In addition, variations in the availability and accessibility of maternal healthcare services, particularly about family planning methods, play a significant role in shaping these outcomes (27).

Multivariable logistic regression analysis showed that the age of the women was associated with LACM uptake. This result is consistent with those of prior studies in many countries (15, 28). This could be the result of younger women favoring short-acting methods of contraception for spacing or delaying their pregnancies, while older women have completed their fertility. Moreover, a possible explanation for this is that older women tend to have more children, have completed their family size, and perhaps do not want more children; hence their preference for LACMs instead of short-acting contraceptives (32).

Media exposure has emerged as a strong predictor of the utilization of LACMs among reproductive-aged women. The likelihood of using LACMs was considerably higher among women who reported having come across FP messages via newspapers, television, or radio in previous months than among those who did not. This can be partly explained by the fact that the media is effective in disseminating information, which increases awareness about healthcare information and healthcare facilities that are available and fosters inter-personnel communication, which could facilitate behavioral changes (33).

Partner’s education was one of the most significant criteria that positively correlated with the intention to use LACMs. Likewise, this had a favorable correlation with the use of contraception in the Butajira district (34) in a systematic review and meta-analysis carried out in Ethiopia (35). Education might help the discussion on modern contraceptives and would increase knowledge about modern FP methods and therefore increase predisposition to their intention and use of LACMs. Women with higher levels of education and supportive partners are more likely to use LACMs, reflecting the need for targeted educational campaigns that address misconceptions and promote the benefits of these methods (36).

Regarding the household wealth quintile, the women who belonged to households in the highest wealth quintile were less likely to use LACMs compared to women who belonged to the lowest wealth quintile. This result is consistent with studies conducted in Ethiopia (37), Nigeria (38), and Malawi (39). This might be because women in lower wealth quintiles have less access to information about LACMs. People's socioeconomic standing, particularly their ability to obtain contemporary healthcare and education, can be impacted by wealth inequality (40). This study demonstrated a strong correlation between women's occupation and their usage of long-acting contraceptives, which is consistent with previous findings (33). The plausible reason for this is that women who are employed are likely to be more educated and afford LACMs (32).

A multivariable logistic regression analysis revealed a relationship between women's marital status and their use of LACMs. As a result, married women were less likely than single women to adopt LACMs. This evidence is supported by other studies (27, 41). If women are not married, the source of the household’s income comes from one individual only. As a result, she may not be able to afford the cost of rearing children. Hence, these factors may force unmarried women to use long-term and effective methods of contraception compared to their married counterparts. The use of LACMs is also influenced by the desire for fertility in the future. Women who wanted to stop having children were more likely to utilize LACMs compared with women who wanted more children. This finding was supported by research carried out in South Africa (42), Malawi (43), and Ethiopia (44). This may be because women who want to stop fertility may choose long-term and effective methods of contraception from the options available to them (41).

The findings of the present study indicate that community-level factors have an impact on the utilization of LACMs. Compared with women who lived in rural regions, urban women were 1.23 times more likely to use LACMs. This evidence is supported by other studies carried out in different countries, such as Indonesia (45), Zambia (46), and Kenya (47). These findings may indicate a lack of qualified providers in rural locations, which could be explained by the fact that LACMs rely on provider skills because they require insertions and removals (47). Thus, the results highlight how crucial it is to address healthcare provider capacity, especially in rural regions, to increase the use of LACMs.

### Strengths and limitations

This study was a pooled analysis, which increases the study's power by allowing for a more in-depth investigation of impact modification in the data and a decrease in measurement errors and bias that can happen when studies with various designs and data collection techniques are combined. This study may not establish a causal relationship between the outcome variable and independent variables due to the cross-sectional nature of the study design. In addition, this study used proxy variables, such as service access and other cluster-level parameters, so they might not correctly reflect the actual situation. The DHS relies on respondents’ self-reporting and may be prone to recall bias. Furthermore, datasets were utilized from hugely different periods to assess the pooled prevalence, which may have influenced the estimated result.

## Conclusion

This study adds to the corpus of research showing how crucial it is to look at community influences on contraceptive behavior to comprehend how factors other than those affecting an individual or household might affect contraceptive practice. The findings of this study indicate that the demand for long-acting reversible and permanent contraception services in East Africa is influenced not only by women's individual and household socioeconomic characteristics and exposure to and accessibility to family planning information and services. The overall utilization of long-acting contraceptive methods was low. In the multivariable mixed-effect binary logistic regression analysis, factors significantly and positively associated with LACM uptake in East African countries included older age, being currently married, tertiary education level of both women and their husbands, current employment, higher wealth index, media exposure, a preference for no additional children, living in urban areas, the absence of significant distance barriers to health facilities, and country of residence. Therefore, future interventions to improve LACM uptake should be planned to target women who are younger, from lower socioeconomic backgrounds, and live in rural areas.

## Data Availability

Publicly available datasets were analyzed in this study. These data can be found here: https://dhsprogram.com/data/available-datasets.cfm.
